# SOCIODEMOGRAPHIC DIFFERENCES IN ADVANCE CARE PLANNING AMONG OLDER LESBIAN AND GAY ADULTS

**DOI:** 10.1093/geroni/igad104.2370

**Published:** 2023-12-21

**Authors:** Meki Singleton, Susan Enguidanos

**Affiliations:** University of Southern California, Leonard Davis S, Los Angeles, California, United States; University of Southern California, Los Angeles, California, United States

## Abstract

Advance care planning (ACP) is a process that supports adults at any stage of health in ensuring that their values, goals, and wishes are met regarding future medical care. Factors associated with completing ACP include older age, being White, higher education, and higher socioeconomic status. However, scant research has investigated if these same characteristics are associated with ACP among lesbian and gay adults. Using data from an online survey (N=139), bivariate analyses were conducted to investigate predictors of ACP outcome measures. Being White (p<.01), older (p<.01), and employed (p<.05) were associated with having a higher level of comfort with medical decision making. Having a higher income (p<.01), being employed (p<.01), and having a health condition (p<.05) were associated with having completed a living will. Having a higher income (p<.01) and being employed (p<.05) were associated with having a durable power of attorney for healthcare. These outcomes demonstrate that while some variables were similar to previous findings, additional sociodemographic characteristics were found to have an influence on ACP among older lesbian and gay adults. Given that completing ACP has shown to improve quality of care at end of life, it is imperative to understand what factors influence ACP for older lesbian and gay adults. Understanding these within group differences will lead to future research and outreach efforts developed to inform and equip healthcare workers and community organizations with the means to better serve older gay and lesbian adults, a group that has been historically discriminated against by the healthcare system.

